# Chinese Giant Salamander (*Andrias davidianus*) Iridovirus Infection Leads to Apoptotic Cell Death through Mitochondrial Damage, Caspases Activation, and Expression of Apoptotic-Related Genes

**DOI:** 10.3390/ijms20246149

**Published:** 2019-12-05

**Authors:** Yiqun Li, Nan Jiang, Yuding Fan, Yong Zhou, Wenzhi Liu, Mingyang Xue, Yan Meng, Lingbing Zeng

**Affiliations:** Yangtze River Fisheries Research Institute, Chinese Academy of Fishery Sciences, Wuhan 430223, China; liyq@yfi.ac.cn (Y.L.); jn851027@yfi.ac.cn (N.J.); fanyd@yfi.ac.cn (Y.F.);

**Keywords:** Chinese giant salamander iridovirus, apoptosis, caspases activation, mitochondria, p53

## Abstract

Chinese giant salamander iridovirus (GSIV) is the causative pathogen of Chinese giant salamander (*Andrias davidianus*) iridovirosis, leading to severe infectious disease and huge economic losses. However, the infection mechanism by GSIV is far from clear. In this study, a Chinese giant salamander muscle (GSM) cell line is used to investigate the mechanism of cell death during GSIV infection. Microscopy observation and DNA ladder analysis revealed that DNA fragmentation happens during GSIV infection. Flow cytometry analysis showed that apoptotic cells in GSIV-infected cells were significantly higher than that in control cells. Caspase 8, 9, and 3 were activated in GSIV-infected cells compared with the uninfected cells. Consistently, mitochondria membrane potential (MMP) was significantly reduced, and cytochrome c was released into cytosol during GSIV infection. p53 expression increased at an early stage of GSIV infection and then slightly decreased late in infection. Furthermore, mRNA expression levels of pro-apoptotic genes participating in the extrinsic and intrinsic pathway were significantly up-regulated during GSIV infection, while those of anti-apoptotic genes were restrained in early infection and then rose in late infection. These results collectively indicate that GSIV induces GSM apoptotic cell death involving mitochondrial damage, caspases activation, p53 expression, and pro-apoptotic molecules up-regulation.

## 1. Introduction

Apoptosis, pyroptosis, and necroptosis are the three major ways of programmed cell death following virus infection, and apoptosis is the most extensively studied programmed cell death during the process [1,2,3]. Hosts eliminate virally infected cells via apoptosis; similarly, some viruses induce apoptosis to release and disseminate their progenies [4]. Apoptosis can be triggered by two fundamentally distinct signaling cascades, namely the extrinsic pathway (death adaptor pathway) and the intrinsic pathway (mitochondrial pathway) [5,6]. The extrinsic pathway is started by the ligand-induced oligomerization of specific cell surface receptors, and results in the activation of caspase 8 and then caspase 3, leading to apoptosis [5]. The intrinsic pathway is controlled by mitochondria, and the signals come from other organelles as well as the extracellular microenvironment [7]. The molecular mechanisms and signal pathways of virus-induced apoptosis have been disclosed in virus infection of mammal cells. It has been reported that porcine circovirus type 2 induces B lymphocyte depletion through apoptosis [8]. Canine parvovirus NS1 can induce apoptosis proceeds through the intrinsic pathway which involves mitochondria, accumulation of reactive oxygen species, and activation of caspases [9]. In chronic hepatitis C virus infection, enhanced hepatocyte apoptosis and up-regulation of the death receptors have been described [10]. In human astrocytes, enterovirus 71 infection led to intrinsic apoptosis and induction of p38-regulated pro-inflammatory cytokines [11]. The tumor suppressor p53 is also employed by host cells during apoptosis induced by virus [12]. Previous studies have indicated that p53 contributes to inhibit the spread of the virus by the activation of apoptosis [13,14]. However, the molecular mechanism of viral infection in lower vertebrates like amphibians remains unclear.

Iridovirus belongs to the genus *Ranavirus* in the family Iridoviridae that displays icosahedral symmetry with a 120–200 nm diameter [15]. It has been reported recently that iridoviruses are able to infect lower vertebrates and invertebrates, leading to huge economic losses in aquaculture [16,17,18]. Iridoviruses, including epizootic hematopoietic virus, frog virus 3, red sea bream iridovirus, lymphocystis disease virus, and Chilo iridescent virus can induce apoptosis in their host cells [19,20,21,22,23]. A previous study showed that giant seaperch iridovirus induces apoptotic cell death through up-regulation of the pro-apoptotic genes Bax and Bak in a grouper fin cell line [17]. Mitochondrion-mediated apoptosis and mitochondrial fragmentation occurred during Rana grylio virus infection in fish cells [24]. The largemouth Bass virus infection also induced the PI3K and ERK signaling pathways involved in apoptosis [25].

Chinese giant salamander (*Andrias davidianus*), one of the largest amphibian species in the world, is of great evolutionary importance and classified as a critically endangered species by the International Union for Conservation of Nature and Natural Resources [26]. In recent years, the Chinese giant salamander has suffered from a severe iridovirosis caused by the Chinese giant salamander iridovirus (GSIV) [27,28]. GSIV belongs to the family Iridoviridae and is a double-stranded DNA virus with icosahedral symmetry and a size of approximately 140 nm in diameter [26]. Continuous studies have been done to explore the infection pathways and immune responses during a GSIV invasion. Transcriptomic analysis of host response to GSIV infection in Chinese giant salamanders has provided new insights on the functions of immune genes and the interactions of the virus and the host [29]. Chen [30] reported that Chinese gaint salamander IFN played an important role in anti-GSIV innate immune response. It is reported that autophagy and apoptosis are activated in Chinese giant salamander immune organs during the early stage of GSIV infection [31].

In the present study, the form of GSIV-induced cell death as well as its regulatory pathways in a Chinese giant salamander muscle cell line (GSM) are investigated. Our results indicate that GSIV infection evokes typical apoptosis by both extrinsic and intrinsic pathways, with mitochondrial changes, caspases activation, p53 expression, and pro-apoptotic factors up-regulation. Our findings may contribute to elucidate the mechanism of GSIV infection.

## 2. Results

### 2.1. Cytopathic Effect

**Figure 1:**
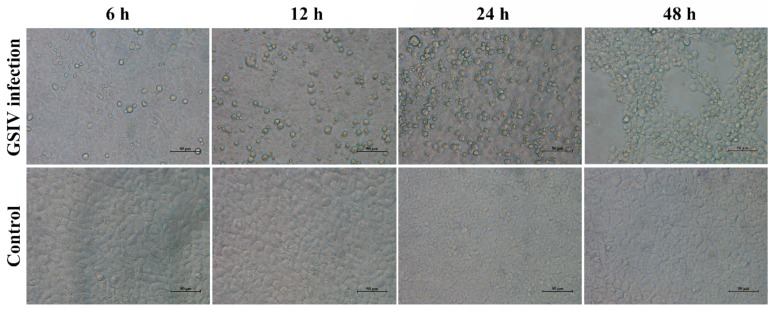
Morphology changes in GSM cells after infected with GSIV (MOI = 0.5) for 6, 12, 24, and 48 h. GSM cells without GSIV infection were set as control. Scar bar, 50 µm.

Microscopy observation showed that GSIV induced host cell death gradually within 48 hours (Figure 1). Compared with control cells, GSIV-infected cells exhibited visible cell death at 6 h p.i., with reduced cell volume and round in shape. Increased cell death was found in GSIV-infected cells at 12 and 24 h p.i., and at 48 h p.i., when apparent cytopathic effect arose. ddPCR analysis showed that the copies of GSIV major caspid protein (MCP) sharply increased from 6 h p.i. (Figure S1).

### 2.2. DNA Fragmentation

**Figure 2:**
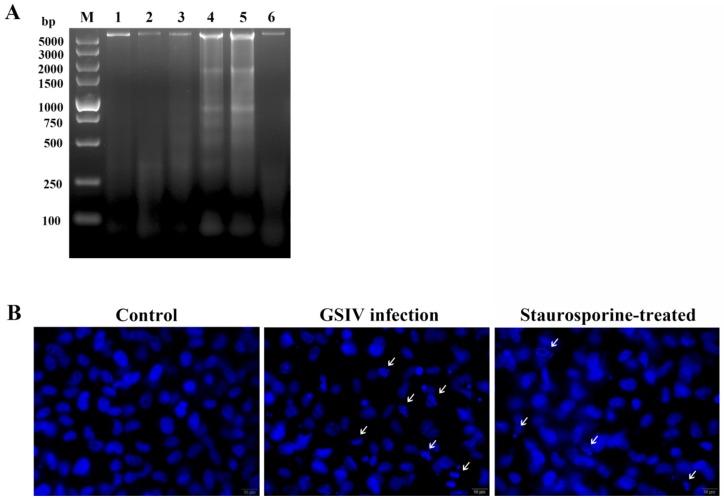
DNA fragmentation in GSM cells during GSIV infection. (**A**) DNA ladder assay at different times after infection (MOI = 0.5). M, DNA marker; 1, 0 h p.i.; 2, 6 h p.i.; 3, 12 h p.i.; 4, 24 h p.i.; 5, 48 h p.i. 6, staurosporine treated for 30 min. (**B**) Nucleus staining after infected with GSIV (MOI = 0.5) for 24 h. GSM cells without GSIV infection were set as control. GSM cells treated with staurosporine were set as positive control. White arrows indicate fragmental nuclei. Scar bar, 10 µm.

DNA fragmentation was examined in GSIV-infected cells. A DNA ladder assay revealed that DNA fragmentation begins at 6 h p.i. which was similar to staurosporine treated cells, and fragmentation intensity increased along with the infection time (Figure 2A). Furthermore, as shown in Figure 2B, as with staurosporine treated cells, nucleus fragmentation was observed in GSM cells at 24 h post-infection (p.i.), while control cells possessed uniformly stained nuclei. To further confirm whether the indicated cells are virus infected, a monoclonal antibody of GISV MCP was used to detect the GSIV distribution. Results indicated that nucleus fragmentation was found in MCP positive cells (Figure S2).

### 2.3. Typical Apoptotic Cell Death in GSM Cells During GSIV Infection

**Figure 3:**
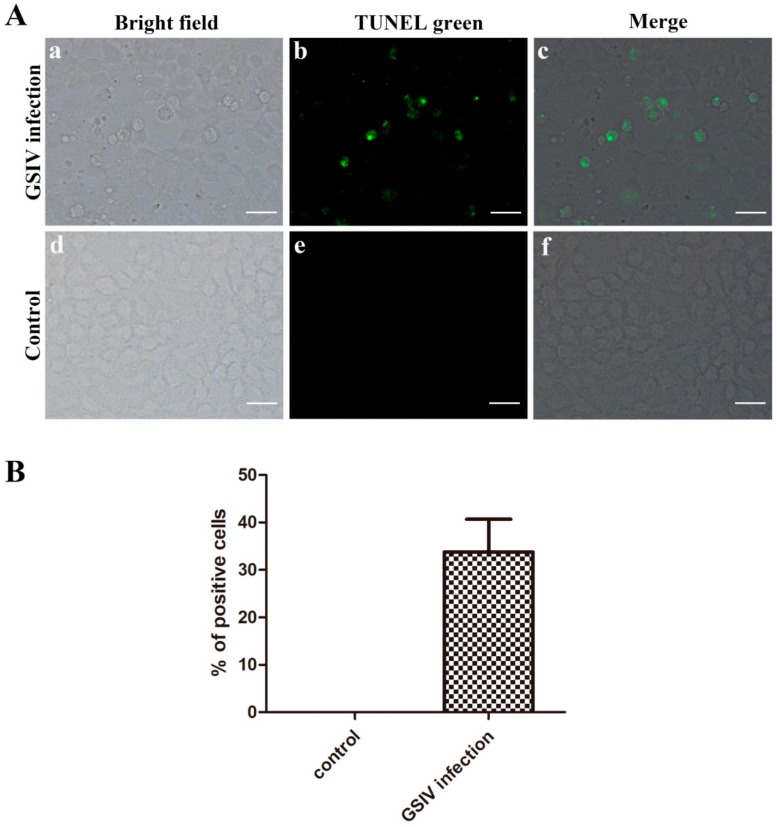
Apoptosis analysis of GSM cells after infected with GSIV (MOI = 0.5) for 24 h by TUNEL detection. (**A**) GSM cells without GSIV infection were set as control. c, a merged image of a and b; f, merged image of d and e. Scar bar, 10 µm. (**B**) Percentage of TUNEL positive cells. Data are obtained from three independent experiments.

**Figure 4:**
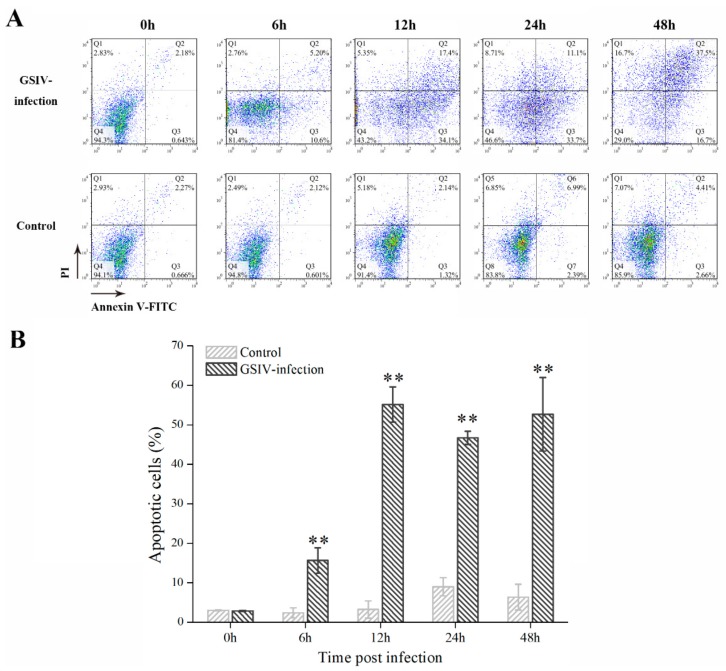
Apoptosis analysis of GSM cells after infected with GSIV (MOI = 0.5) by flow cytometry. (**A**) FACS analysis of GSM cells treated with or without GSIV and stained annexin V-FITC and PI. (**B**) Rate of apoptotic cells with or without GSIV infection. Error bars represent as mean ± SD; ** *p* < 0.01. All data shown are reproducible and representative of three independent experiments.

A TUNEL assay showed that positive signals (green) were observed in GSM cells with GSIV infection (Figure 3A), which indicated that GSM cells with GSIV infection underwent apoptosis. The percentage of TUNNEL positive cells is 33.78% ± 6.92% (Figure 3B). In contrast, no positive signal was found in control GSM cells. To further qualified the percentage of apoptotic cells, GSM cells with or without GSIV infection were stained with annexin V and PI and analyzed by flow cytometry (Figure 4A). The cells were gated according to the cells unstained as Figure S3. The result indicated that the proportion of apoptotic cells (the sum of PI-/annexin V+ cells and PI+/annexin V+ cells) in GSIV-infected cells was increased from 15.65% ± 1.19% at 6 h p.i. to 52.57% ± 9.30% at 48 h p.i., which was significantly (*p* < 0.01) higher than that in control cells (Figure 4B).

### 2.4. Caspases Activation

**Figure 5:**
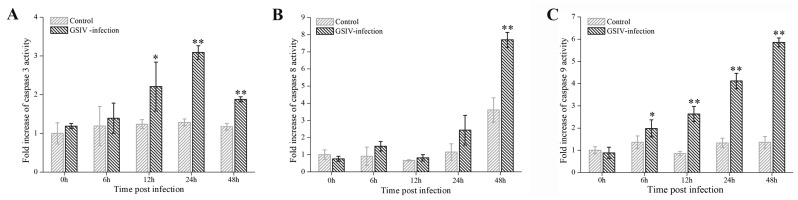
Caspases activity induced by GSIV infection at indicated time points. Caspase 3 (**A**), 8 (**B**), 9 (**C**) activities were determined using fluorescein active caspase staining kit by flow cytometry. Data are obtained from three independent experiments. Error bars represent as mean ± SD; * *p* < 0.05, ** *p* < 0.01.

To investigate whether caspases were activated during GSIV infection, the activities of caspase 3, caspase 8 and caspase 9 were examined by flow cytometry and the samples were gated according to cells unstained as Figure S4. As shown in Figure 5A, caspase 3 activity in GSIV-infected cells significantly (*p* < 0.05) increased (about 2.2-fold) at 12 h p.i. compared to that in control cells, and reached a peak level of 3.1-fold at 24 h p.i. Activity of caspase 8 didn’t increase till 48 h p.i., which rose up to about 7.7-fold in comparison to control cells (Figure 5B). Furthermore, caspase 9 activity in GSIV-infected cells increased significantly (*p* < 0.05, 2-fold) as early as 6 h p.i., continuously rose up at 12 h (2.6-fold) and 24 h p.i. (4.2-fold), and peaked at 48 h p.i. (5.9-fold) in comparison to that in control group (Figure 5C).

### 2.5. Mitochondrial Membrane Potential (MMP)

**Figure 6:**
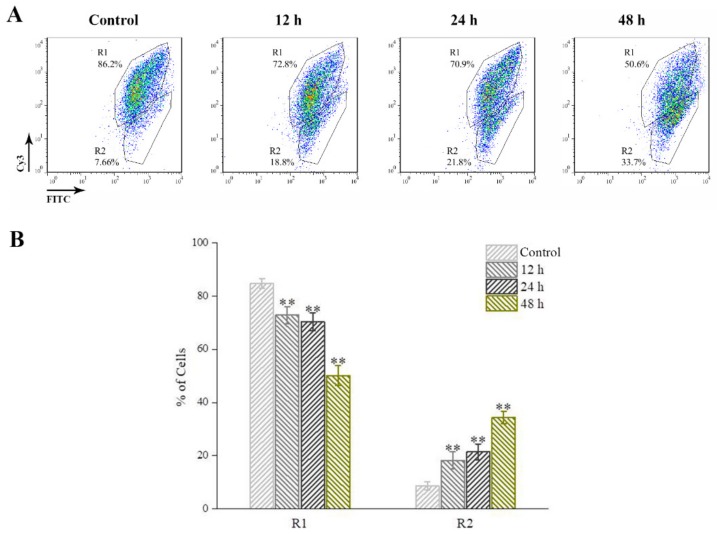
GSIV infection reduced mitochondrial membrane potential (MMP). (**A**) FACS analysis of GSM cells treated with GSIV for 12, 24, and 48 h and stained with JC-1. Cells without GSIV infection was set as control. (**B**) Change in the percentage of cells in R1 and R2 with or without GSIV infection. Data are obtained from three independent experiments. Error bars represent as mean ± SD; ** *p* < 0.01.

JC-1 dye is used to detect the MMP during GSIV infection. The depolarization of MMP accompanied with the reduced red fluorescent signals. The flow cytometry based on Cy3 fluorescence and FITC fluorescence revealed that there are two cell population, designed as R1 (with stronger red fluorescent signals) and R2 (with weaker red fluorescent signals). GSIV infected GSM cells exhibited weakened red fluorescent signals (Figure 6A) compared with control cells. The percentage of cells with reduced MMP in GSIV infected group at 12 h p.i. significantly (*p* < 0.01) increased to 18.3% ± 3.2% and further increased up to 21.5% ± 2.9% and 34.4% ± 2.3% at 24 h and 48 h p.i., respectively, in compared to that in control group (Figure 6B).

### 2.6. Cytochrome c Release

**Figure 7:**
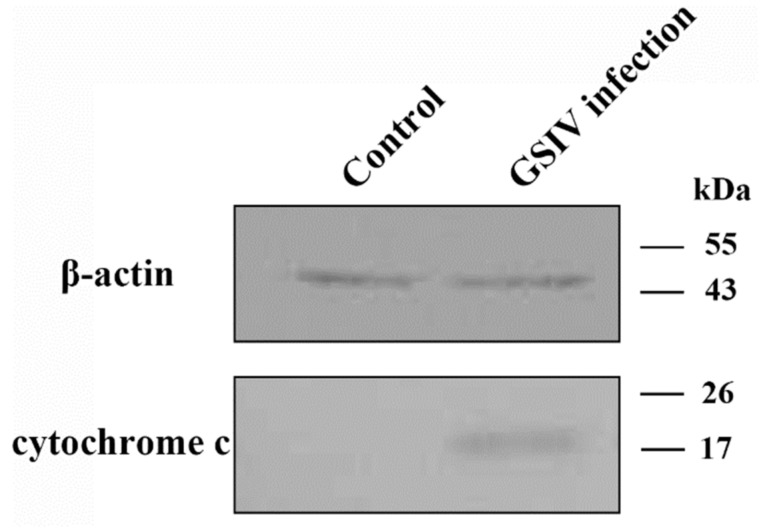
Analysis of cytochrome C release under GSIV infection for 24 h. Cytosolic proteins were extracted from GSM cells with or without GSIV infection for 24 h, and analyzed by Western blot. β-actin was treated as a reference.

Loss of MMP results in membrane permeabilization and cytochrome c release. To investigate whether cytochrome c release occurred in GSM cells during GSIV infection, cytosolic proteins of GSIV infected cells and control cells at 24 h p.i. were extracted to detect the presence of cytochrome c by Western blot. The results exhibited that cytochrome c was found in cytosolic proteins of GSIV infected cells (Figure 7). In contrast, cytochrome c was undetectable in cytosolic proteins of control cells.

### 2.7. p53 Expression

**Figure 8:**
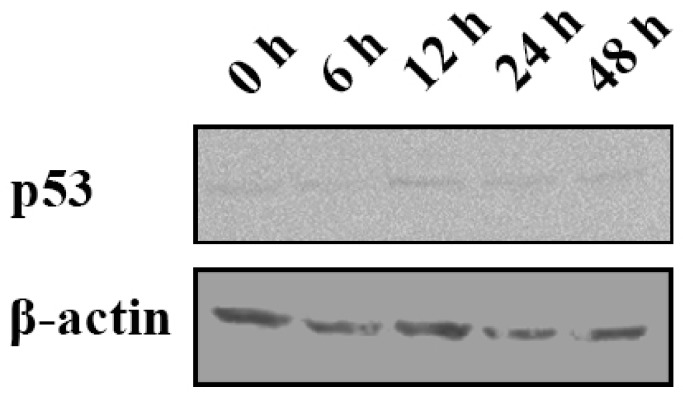
GSIV influences p53 expression in GSM cells. Proteins were extracted from GSM cells GSIV infection at indicated time points, and analyzed by Western blot. β-actin was treated as a reference.

To clarify whether p53 participated in this process, p53 expression was detected by Western blot. The results showed that expression of p53 protein kept a low level at the beginning, then increased at 12 h post GSIV infection, and slightly decreased at 24 h and 48 h p.i. (Figure 8).

### 2.8. mRNA Expression of Apoptosis Associated Genes

**Figure 9:**
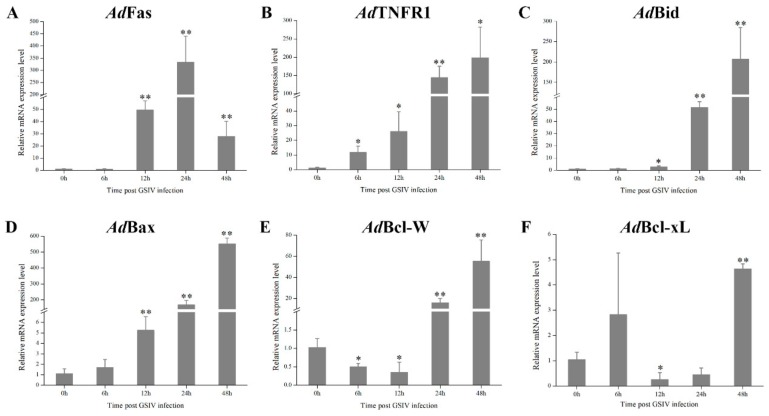
mRNA expression patterns of genes. (**A**) *Ad*Fas, (**B**) *Ad*TNFR1, (**C**) *Ad*Bid, (**D**) *Ad*Bax, (**E**) *Ad*Bcl-W, and (**F**) *Ad*Bcl-xL participated in extrinsic and intrinsic pathways after GSIV infection at indicated time points. For convenience of comparison, the expression level at 0 h was set as 1. Data are obtained from three independent experiments. Error bars represent as mean ± SD; * *p* < 0.05, ** *p* < 0.01.

The expression level of AdFas increased significantly (*p* < 0.01) at 12 h p.i. and peaked at 24 h p.i. (Figure 9A). Similarly, the expression levels of AdTNFR1, AdBid and AdBax continuously up-regulated from 6, 12, and 12 h p.i., respectively (Figure 9B–D). The expression level of AdBcl-W decreased significantly (*p* < 0.05) at 12 h, went back to the original level 24 h p.i. and then increased significantly (*p* < 0.01) at 48 h p.i. (Figure 9E). The expression level of AdBcl-xL down-regulated significantly (*p* < 0.05) from 12 h to 24 h p.i. and then increased from 24 h to 48 h p.i. (Figure 9F).

## 3. Discussion

Apoptosis is an typical outcome following infection by most viruses [32]. For iridovirus, several studies have shown that they can induce apoptosis in their host cells [17,25,32]. Likewise, in our present study, microscopy and flow cytometry revealed typical apoptosis in GSIV-infected GSM cells, accompanied by dramatic reduction in cell volume, DNA fragmentation, and exposure of phosphatydylserine on the outer leaflet of the plasma membrane.

The extrinsic and intrinsic pathways are two distinct signal pathways involved in apoptosis. The extrinsic pathway is started by ligand–ligand oligomerization of specific cell surface receptors, including Fas/CD95 and the tumor necrosis factor receptor (TNFR) [7]. Ligand–ligand oligomerization of the receptors then induce the intracellular assembly of death-inducing signaling complex (DISC), then activation of caspase cascade that emanates from caspase 8 to effector caspases (caspase 3, 6, 7) and nucleases, leading to apoptosis [1,19]. In our study, the mRNA expression levels of *Ad*Fas and *Ad*TNFR1 significantly up-regulated during GSIV infection, indicating specific cell surface receptors in the extrinsic pathway might participate in this process. Moreover, the activities of caspase 8 and caspase 3 in GSIV-infected cells increased significantly compared to that in control cells, suggesting that caspase cascade involved in the extrinsic pathway are activated during GSIV infection. These results together demonstrate that the extrinsic pathway is involved in GSIV-induced apoptosis. The intrinsic pathway is activated by various developmental cues and cytotoxic insults such as viral infection, DNA damage, and growth factor deprivation. This process is controlled by mitochondria which collect and integrate pro- and anti-apoptotic signals from other organelles as well as extracellular microenvironment [33,34]. Pro-apoptotic signals favor mitochondrial membrane permeabilization, followed by the release of mitochondrial intermembrane space proteins including caspase activators (e.g., cytochrome c) into the cytosol, and lead to activation of caspase 9 and finally activate effector caspase 3 and caspase 7 [35]. In current study, we found that the MMP reduced significantly during GSIV infection, and cytochrome c was detected in the cytoplasmic fraction of GSIV-infected cells rather than that of control cells. Consistently, the activities of caspase 9 and caspase 3 in GSIV-infected cells significantly increased compared to that in control cells. Moreover, a cell’s decision to live or die rests largely with the Bcl-2 family of interacting proteins, including both pro-apoptotic molecules (e.g., Bax, Bid, Bak, Bad) and anti-apoptotic molecules (e.g., Bcl-xL, Bcl-W, Mcl-1, Bcl-2) [36]. We found that the mRNA expression levels of *Ad*Bax and *Ad*Bid in GSM cells significantly increased after infected with GSIV, while those of *Ad*Bcl-xL and *Ad*Bcl-W were significantly reduced during the early infection, suggesting that the activity of Bcl-2 family might be regulated during GSIV infection. These results collectively indicate that the intrinsic pathway plays a crucial role during GSIV-induced apoptosis. Nevertheless, the detailed function of the Bcl-2 family in GSIV-induced apoptosis still needs to be further investigated.

It has been reported that p53 participates in the defense against viral infection by activating cell-cycle arrest or apoptosis via the transcription of target genes [37]. p53-dependent apoptosis has been identified as a powerful control to restrict virus infection. In carp, spring viremia of carp virus (SVCV) degraded p53 early in the infection, while an increase of p53 was observed late in the infection [38]. In this study, we found an absolutely inverse phenomenon. The expression of Chinese giant salamander p53 increased at the early stage of GSIV infection and then went down slightly in late infection, which implied that the DNA virus GSIV might guide a different mechanism during p53-dependent apoptosis induced by the virus. The game between host and virus is a complex process, and the regulation of host p53 during GSIV infection will be further studied in the future.

In conclusion, we demonstrate the elementary mechanism of cell death by GSIV infection in the present study. It is apparent that a GSIV infection induces typical apoptosis in GSM cells. Moreover, signals in both the extrinsic and intrinsic pathways are involved in GSIV-induced apoptosis. In addition, p53 also participates in this process. These results provide new insights into the regulatory mechanism of GSIV infection and contribute to find the potential strategy for preventing iridovirus infection.

## 4. Materials and Methods

### 4.1. Cell Culture and Virus

The giant salamander muscle (GSM) cell line was generously presented by Prof. Qiya Zhang, Institute of Hydrobiology, Chinese Academy of Sciences, Wuhan and grown in medium 199 (Hyclone, Logan, UT, USA) supplemented with 10% fetal bovine serum at 20 °C. The GSIV was originally isolated and identified from diseased Chinese giant salamanders by our laboratory [28].

### 4.2. Droplet Digital PCR (ddPCR) of GSIV MCP Copy

At 0, 6, 12, 24, and 48 h post GSIV infection (MOI = 0.5), GSM cells were harvested and washed with PBS. Virus DNA were isolated with Viral DNA Kit (Omega Bio-tek, Norcross, GA, USA) according to the manufactures’s protocols. ddPCR was performed in an QX200TM Droplet Digital PCRTM system (Bio-Rad, Hercules, CA, USA) using QX200 ddPCR EvaGreen Dye Kit (Bio-Rad, Hercules, CA, USA) with primers MCP-F (5’-GCGGTTCTCACACGCAGTC-3’) /MCP-R (5’-ACGGGAGTGACGCAGGTGT-3’). The ddPCR mixture consisted of 2 μL of the diluted cDNA sample, 10 μL 2 × QX200 ddPCR EvaGreen^®^ Supermix, 0.2 μL of each primer (10 M) and 7.6 μL H_2_O. The ddPCR cycle profile included 1 cycle at 95 °C for 5 min, then 40 cycle at 95 °C for 30 s, 55 °C for 1 min, then 4 °C for 5 min, 90 °C for 5 min.

### 4.3. Nucleus Staining

GSM cells were grown on 20-mm circular coverslips and cultured in 12-wells for 24 h. Then GSIV was inoculated onto GSM cells at a multiplicity of infection (MOI) of 0.5 for 24 h, and GSM cells without GSIV infection were set as control and with staurosporine-treatment (30 min) were set as positive control. The cells were fixed with 4% paraformaldehyde for 20 min and washed three times with PBS. The cells were stained with DAPI (10 µg/mL) (Solarbio, Beijing, China) for 10 min at room temperature in the dark. After washing as above, the cells were observed with a fluorescent microscope (Olympus, Tokyo, Japan).

### 4.4. DNA Fragmentation Assay

The confluent monolayer of GSM cells were infected with GSIV at a MOI of 0.5. At 6, 12, 24, and 48 h post-infection, the infected GSM cells and the control were harvested and the total genomic DNA was extracted as reported previously [24]. staurosporine-treated (30 min) GSM cells was as a positive control. Briefly, the collected cells were lysed with DNAzol (Invitrogen, Carlsbad, CA, USA) and the lysate was centrifuged at 12,000 ×*g* for 5 min. Then the supernatant was mixed with equivalent 100% (*v/v*) ethanol and transferred to an separation column. After washed with 70% ethanol and dried, DNA was dissolved in double-distilled water and then analyzed by 2% agarose gel electrophoresis.

### 4.5. TUNEL Assay

An in situ cell death detection kit (Roche, Mannheim, Germany) was used to further analyze apoptosis based on labeling of DNA strand breaks. After grown on 20-mm circular coverslips for 24 h, GSM cells were infected with or without GSIV at a MOI of 0.5 for another 24 h. The cells were fixed with 4% PFA for 1 h at room temperature and permeabilized with 0.1% Triton X-100 for 2 min on ice. After rinsed twice with PBS, the cells were incubated with TUNEL reaction mixture in above detection kit for 1 h at 37 °C in the dark and then rinsed three times with PBS. The cells were observed with a fluorescent microscope (Olympus, Tokyo, Japan).

### 4.6. Flow Cytometry

#### 4.6.1. Flow Cytometry Assay for Apoptosis Detection

At 6, 12, 24, and 48 h post GSIV infection (MOI = 0.5), the infected GSM cells and the control were harvested and washed with PBS. Cells were then suspended with 210 µL staining solution containing annexin V-FITC and PI (Beyotime, Shanghai, China) and incubated at room temperature for 20 min in the dark. After washing, the fluorescence signals were determined by a FACScan flow cytometry (Beckman Coulter, Atlanta, GA, USA). FlowJo 7.6.1 was used for flow cytometric analysis.

#### 4.6.2. Flow Cytometry Assay for Mitochondrial Membrane Potential (MMP) Detection

To explore the function of mitochondrion during GSIV infection, JC-1 was used to evaluate the alteration of mitochondrial membrane potential according to the manufacturer’s protocols. GSM cells were cultured in 24-wells plate for 24 h, and then infected with GSIV at a MOI of 0.5. At 12, 24, and 48 h post-infection, GSM cells were harvested, washed and resuspended in fresh culture medium. The cells were incubated with JC-1 dye solution (Beyotime, China) for 20 min. After washed three times, the cells were resuspended in JC-1 buffer and quantified by flow cytometry (Beckman Coulter, Atlanta, GA, USA). FlowJo 7.6.1 was used for flow cytometric analysis.

#### 4.6.3. Flow Cytometry Assay for Caspases Activity

The activities of caspase 3, 8, and 9 were detected using a fluorometric protease assay kit (BioVision, San Francisco, CA, USA) following the manufacturer’s instruction. Briefly, GSM cells were cultured in 24-wells plate for 24 h, and then infected with GSIV at a MOI of 0.5. Then infected and control GSM cells were harvested at 0, 6, 12, 24, and 48 h post-infection and incubated with FITC-DEVD-FMK (for caspase 3 detection), FITC-IETD-FMK (for caspase 8 detection), or FITC-LEHD-FMK (for caspase 9 detection) at 20 °C for 1 h, separately. After washed three times with wash buffer, samples were analyzed by flow cytometry (Beckman Coulter, Atlanta, GA, USA). FlowJo 7.6.1 was used for flow cytometric analysis.

### 4.7. Western Blot

#### 4.7.1. Western Blot Analysis for Cytochrome c Release

Samples of cytosolic proteins of GSM cells with or without GSIV infection (MOI of 0.5) were prepared using a cell mitochondria isolation kit (Beyotime, China) following the manufacturer’s instruction. Proteins were separated by 12% SDS-PAGE and then electro-transferred to 0.22 µm PVDF membranes with a semi-dry blotter (Bio-Rad, Hercules, CA, USA). The membranes were blocked subsequently in TBST (0.1% Tween-20, pH 7.5) containing 5% skim milk at room temperature for 2 h. The membranes were then respectively incubated with an anti-cytochrome c rabbit polyclonal antibody (Abclonal, Boston, MA, USA) at a dilution of 1:500, and anti-β-actin antibody (CST, Danvers, MA, USA) at a dilution of 1:1000 at 4 °C overnight. After three times of washing with TBST, the membranes were respectively incubated with HRP-labeled goat-anti-rabbit IgG (Invitrogen, Carlsbad, CA, USA) and goat-anti-mouse IgG (Invitrogen, Carlsbad, CA, USA) for 1 h. After rinsed with TBST for three times, the membranes were incubated in Western lighting-ECL substrate system (Perkin Elmer) before exposure to a ChemiDoc^TM^ XRS+ imaging system (Bio-Rad, Hercules, CA, USA). β-actin was treated as a reference.

#### 4.7.2. Western Blot Analysis for p53 Expression

Protein samples of GSM cells with GSIV infection (MOI = 0.5) for different time points were prepared as above. After separated by SDS-PAGE, transferred to PVDF membranes and blocked, the membranes were then respectively incubated with an anti-p53 mouse antibody (CST, Danvers, MA, USA) at a dilution of 1:1000, and anti-β-actin antibody (CST, Danvers, MA, USA) at a dilution of 1:1000 at 4 °C overnight. After washed and incubated with HRP-labeled goat-anti-mouse IgG (Invitrogen, Carlsbad, CA, USA), the membranes were detected as above. β-actin was treated as a reference.

### 4.8. Quantitative Real-Time PCR (qRT-PCR) of Apoptosis Related Genes Expression

qRT-PCR was performed as reported previously [39]. Briefly, GSM cells (at 0, 6, 12, 24, and 48 h post GSIV infection, at a MOI of 0.5) were harvested aseptically from four 24-wells and used for total RNA extraction with EZNA total RNA kit (Omega Bio-tek, Norcross, GA, USA). After treated with RNase-free DNase I (TaKaRa, Taejin, Japan), the quality of the RNA was examined by determining 260/280 absorbance ratio using NanoDrop One (Thermo scientific, Waltham, MA, USA) and by gel electrophoresis. Reverse transcription of cDNA was performed according to the protocol of the ReverlAid First Stand cDNA synthesis kit (Thermo Scientific, Waltham, MA, USA). qRT-PCR was carried out in an Roter-Gene Q PCR system (Qiagen, Dusseldorf, Germany) using SYBR Green dye kit (Toyobo, Osaka, Japan). The PCR reaction was performed in a volume of 20 µL reaction mixture containing 10 µL 2 × SYBR Green premixture, 1 µL diluted template cDNA (300 ng/µL), 0.5 µL of each target gene primer (10 µM) and 8 µL water. qRT-PCR was performed with the following program: 95 °C for 30 s, then 40 cycles of 95 °C for 15 s, 60 °C for 20 s, and 72 °C for 35 s, followed by an extension at 72 °C for 10 min. The mRNA levels of genes were analyzed using comparative threshold cycle method (2^−ΔΔCT^) with β-actin as an internal reference. The primers designed for qRT-PCR were as follows: *Ad*Fas-F: 5′-AAAGGCAGTCTGAATGTCCA-3′, *Ad*Fas-R: 5′-TGGCAAATGGTATTCGCTTC-3′; *Ad*TNFR1-F: 5′-GAATGCTCCAATGGGAAACC-3′, *Ad*TNFR1-R: 5′-GCATCTCCCTCCAATAGACC-3′; *A*dBid-F: 5′-CATGAGCTTGTAACTGAGCTG-3′, *Ad*Bid-R: 5′-CAATCTCAGCCAGCTTTTGT-3′; *Ad*Bax-F: 5′-CCCCCAAGGAGGTGTTCTTC-3′, *Ad*Bax-R: 5′-AGTAAAAGAGCGCCACCACA-3′; *Ad*Bcl-W-F: 5′-GGACTTTGTGTGGTACAAGC-3′, *Ad*Bcl-W-R: 5′-TCCTTATTCATGCTCTCGCC-3′; *Ad*Bcl-xL-F: 5′-AGTTTGAACTGAGGTATCGC-3′, *Ad*Bcl-xL-R: 5′-CAATGTTCCCTACGAGTCCC-3′; *Ad*β-actin-F: 5′-TGAACCCAAAAGCCAACCGAGAAAAGAT-3′, *Ad*β-actin-R: 5′-TACGACCAGAGGCATACAGGGACAGGAC-3′.

### 4.9. Statistical Analysis

The results were expressed as the mean ± standard deviation (SD) and statistical analyses were carried out with SPSS 17.0 software (SPSS Inc., Chicago, IL, USA). Comparisons between groups were analyzed with the two-sample Student’s *t* test, and statistical significance was defined as *p* < 0.05 and extremely significant as *p* < 0.01.

## Supplementary Materials

Supplementary materials can be found at https://www.mdpi.com/1422-0067/20/24/6149/s1.

## Abbreviations

GSIV	Chinese giant salamander iridovirus
GSM	Chinese giant salamander muscle cell line
MMP	mitochondria membrane potential
MOI	multiplicity of infection
