# Supplementary material for: Chinese Giant Salamander (Andrias davidianus) Iridovirus Infection Leads to Apoptotic Cell Death through Mitochondrial Damage, Caspases Activation, and Expression of Apoptotic-Related Genes
Source: Int J Mol Sci. 2019 Dec 5;20(24):6149. doi: 10.3390/ijms20246149 (PMC6940751; doi:10.3390/ijms20246149)
Supplement: Supplementary file 1 [file ijms-20-06149-s001.pdf]

Supplementary Materials

1.1. Virus replication detection

To confirm the replication of virus during infection, Droplet Digital PCR (ddPCR) was used to detect copies of GISV major caspid protein (MCP) (Figure S1). The result showed that copies of GISV MCP increased from 6 h post infection. Furthermore, a monoclonal antibody of GISV major caspid protein (MCP) was used to detect whether the indicated cells are virus infected (Figure S2). Results indicated that nucleus fragmentation was found in MCP positive cells.

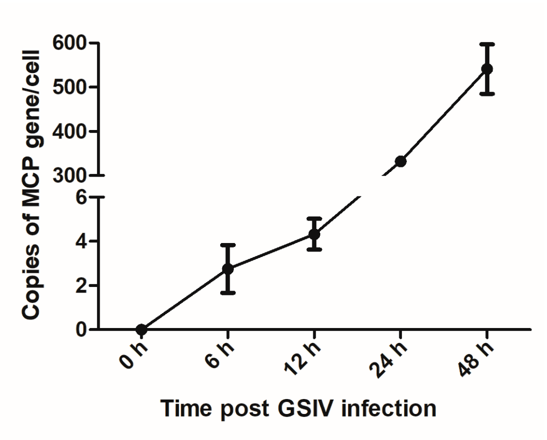

Figure S1. Copies of GISV major caspid protein (MCP) detected by Droplet Digital PCR at 0 h, 6 h, 12 h, 24 h and 48 h post GSIV infection. Data are obtained from three independent experiments. Error bars represent as mean ± SD.

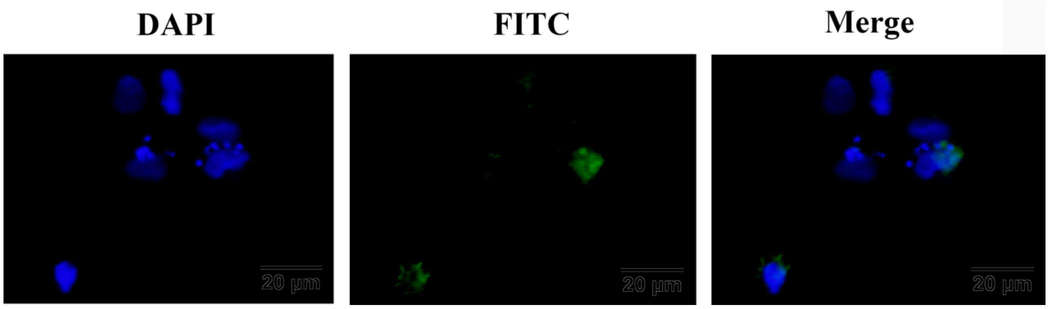

Figure S2. Fluorescence microscopic analysis of GISV major caspid protein (MCP) distribution in GSM cells. After incubated with a monoclonal antibody of GISV major caspid protein (MCP), GSIV-infected GSM cells were then stained with FITC(green color)-labeled goat anti-mouse antibody. Nucleus was stained with DAPI (blue color). Scar bar, 20 μm.

1.2. Gating strategies during flow cytometry analysises

For Annexin V and PI stained cells analysis, GSM cells were gated according to the cells unstained as Figure S3. For caspases activation analysis, the samples were gated according to cells unstained as Figure S4.

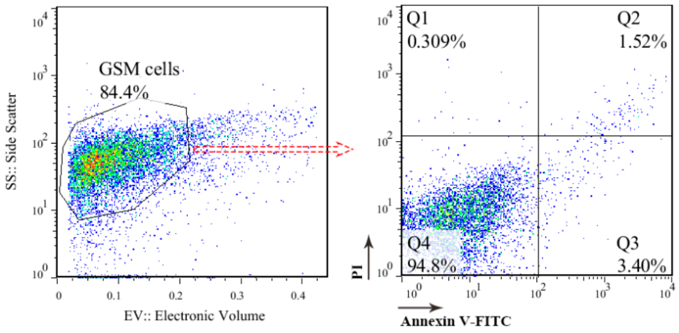

Figure S3. Gating strategy used before annexin V and PI stained cells were analysed

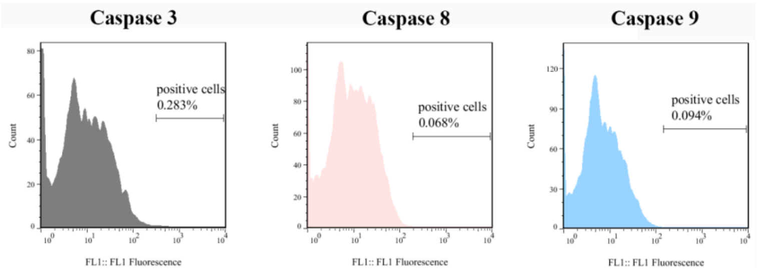

Figure S4. The gating strategy for caspase 3, caspase 8 and caspase 9 activation analysis according to unstained cells
